# Recent Analytical Methods for *N-*Nitrosamine Analysis as Impurities in Drug Substances and Drug Products

**DOI:** 10.1155/jamc/9550913

**Published:** 2026-10-22

**Authors:** Rimadani Pratiwi, Alifa Dhia Shalima, Mutakin, Aliya Nur Hasanah

**Affiliations:** ^1^ Department of Pharmaceutical Analysis and Medicinal Chemistry Faculty of Pharmacy Universitas Padjadjaran Sumedang 45363 Indonesia unpad.ac.id; ^2^ Undergraduate Program of Pharmacy Faculty of Pharmacy Universitas Padjadjaran Sumedang 45363 Indonesia unpad.ac.id

**Keywords:** analysis, drug product, drug substance, impurities, nitrosamine

## Abstract

*N*‐nitrosamine (hereinafter nitrosamine) is among the organic impurities characterized by high potency as genotoxic and probable human carcinogens found in drug substances and drug products. This type of impurity has become a major concern in pharmaceutical industry, since one of nitrosamine type of impurities, NDMA, was detected in valsartan active pharmaceutical ingredients back in 2018. As a result, the development of sensitive, selective, and reliable analytical methods is essential to maintain regulatory compliance and safeguard of patient health. This review provides an overview of recent analytical strategies in 2020–2025 for detecting nitrosamine impurities in pharmaceutical substances and products, with an emphasis on chromatography‐based techniques and groundbreaking analytical approaches. Gas chromatography (GC) and liquid chromatography (LC) coupled with mass spectrometry are the predominant methods, offering high sensitivity and selectivity for quantifying trace levels of these impurities. GC‐based techniques are most appropriate for volatile and thermally stable nitrosamines, whereas LC–MS methods are suitable for polar, nonvolatile, thermally unstable, and structurally complex nitrosamines, including nitrosamine drug substance‐related impurities (NDSRIs). Additionally, alternative LC‐based detectors, such as ultraviolet (UV) and fluorescence detection (FLD), as well as total nitrosamine assays, chemiluminescence, and colorimetric methods, are evaluated as complementary or cost‐effective analytical options. Key analytical considerations, including sample preparation, ionization techniques, matrix effects, derivatization strategies, and method validation, are also examined. Furthermore, this review highlights current challenges and future directions in nitrosamine analysis.

## 1. Introduction

According to the ICH Q3A(R2) guideline, pharmaceutical impurities are classified into organic (process and drug‐related), inorganic, and residual solvents [1]. Among the organic impurities, *N-*nitrosamines (hereinafter nitrosamines) are of particular concern due to the highly potent genotoxicity and are also classified as probable human carcinogens [2]. These compounds are characterized by the presence of a nitroso group (‐N=O) and are regulated under ICH M7 guideline, as long‐term exposure above acceptable limits may increase cancer risk [3, 4]. Even in trace amounts, nitrosamine compounds can react with DNA and cause permanent damage as well as mutagenic effect [5]. Nitrosamines can undergo a sequence of enzymatic reactions, resulting in the production of alkyl diazonium ions (N ≡ N^+^) [6]. The alkylation of DNA by these reactive diazonium ions serves as the molecular trigger for mutagenicity [7]. Their toxicological relevance has been known since early studies indicated carcinogenicity in rats, while further data have connected exposure to liver damage and other deleterious effects in humans and animals [8, 9].

In 2018, *N*‐nitrosodimethylamine (NDMA) was detected in valsartan active pharmaceutical ingredients (APIs) manufactured by Zhejiang Huahai Pharmaceuticals in China, leading the European Medicines Agency (EMA) to issue warning to recall all medicinal products containing that drug substance [10, 11]. This event also prompted expanded recalls of other products, including drugs in the same class, such as losartan and irbesartan [12].

Because of that, regulatory agencies have published acceptable intake (AI) to control nitrosamine impurities in pharmaceutical products and limit prospective carcinogenic risk [13]. The term AI is used to define the intake level at which the health risk is negligible, expressed in nanograms per day (ng/day) [14, 15]. The daily maximum AI of nitrosamine is outlined in ICH M7(R1) guideline as threshold of toxicological concern (TTC). TTC is an exposure limit concept for regulating maximum exposure of nitrosamines, which is not more than 1500 ng/day [16]. However, the method is not applicable to some nitrosamines with more potent genotoxic carcinogens [17]. This shows the need to determine compound‐specific exposure limits through a dedicated carcinogenicity study or a read‐across to an appropriate surrogate compound [16]. Furthermore, regulatory authorities have established a default AI of 18 ng/day for new structures, based on the fifth percentile of nitrosamine compound TD_50_ values from the Lhasa Carcinogenicity Database [18]. The specified AI for nitrosamine impurities is listed in Table 1.

**TABLE 1:** Acceptable intake established for some nitrosamines based on the article 5(3) of regulation (EC) No. 726/2004 referral on nitrosamine impurities in human medicinal products [19–21].

Structure	IUPAC name	Synonym	AI (ng/day)
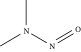	*N*‐Nitroso‐dimethylamine	NDMA	96
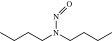	*N*‐Nitroso‐di‐n‐butylamine	NDBA	26.5
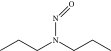	*N*‐Nitroso‐dipropylamine	NDPA	26.5
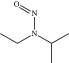	*N-*Nitroso‐ethylisopropylamine	NEIPA	400
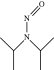	*N*‐Nitroso‐diisopropylamine	NDIPA	1500
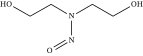	*N*‐Nitroso‐diethanolamine	NDELA	1900
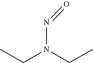	*N*‐Nitroso‐diethylamine	NDEA	26.5
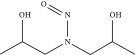	*N*‐Nitroso‐diisopropanolamine	NDIPLA	400
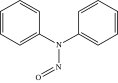	*N*‐Nitroso‐diphenylamine	NDPh	78,000
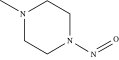	1‐Methyl‐4‐nitrosopiperazine	MeNP	400
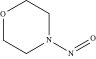	*N*‐Nitroso‐morpholine	NMOR	127
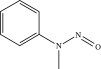	*N*‐Nitroso‐N‐methylaniline	NMPA	100
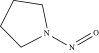	*N*‐Nitroso‐pyrrolidine	NPYR	1700

Although nitrosamines are generally considered chemicals of toxicological concern, their carcinogenic potential is not uniform across the class. Previous evaluations showed that several nitrosamines were noncarcinogenic, highlighting the necessity of compound‐specific risk assessment [22]. In practice, nitrosamine carcinogenicity can be determined using structure‐activity relationship (SAR) models supported by rat TD50 datasets from Carcinogenic Potency Database (CPDB) and Lhasa Carcinogenicity Database (LCDB). These datasets enable relative potency classification, allowing the determination of AI through a surrogate limit analysis when compound‐specific information is unavailable [18].

Despite numerous analytical methods have been developed for detecting nitrosamine impurities, the selection of suitable analytical methods depends on the intended application and sample type. Existing reviews have emphasized nitrosamine toxicology and regulatory developments, such as the history, potential cause, and mitigation strategies. Accordingly, this review summarizes the latest nitrosamine analysis methods from 2020 to 2025, outlines major considerations for selecting appropriate approaches, and discusses emerging trends and future analytical needs.

## 2. Analytical Challenges of Nitrosamines

### 2.1. Formation and Sources of Nitrosamines

Primarily, nitrosamine is formed when secondary, tertiary, or quaternary amine combines with nitrosating substances such as nitrite salts like sodium nitrite (NaNO_2_) under acidic conditions or elevated temperature [15, 23]. In general, nitrosamine formation requires three key elements: a nitrosatable amine, a nitrosating agent, and favorable reaction conditions [24]. The nitrosatable amine can be present either as moieties of drug substance or as impurities [25]. Nitrosation occurs when an amine is present in the unprotonated form, facilitating the formation of the *N*‐nitroso functional group [26]. A general scheme for nitrosamine formation is illustrated in Figure 1. The presence of nitrosamines in the drug can be explained by various root causes attributed to the manufacturing process, as shown in Figure 2.

**FIGURE 1:**
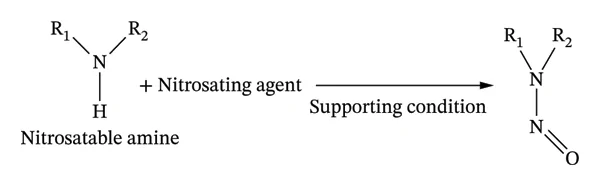
Formation of nitrosamine impurity.

**FIGURE 2:**
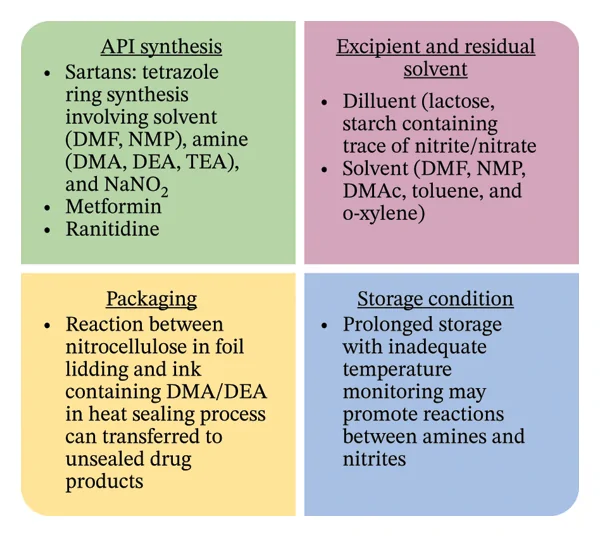
Root cause of nitrosamines in the API and the drug product.

Nitrosamine formation is inseparable from the lifecycle of drugs. A large proportion of known APIs and API‐related impurities have been identified as potential nitrosamine precursors due to the presence of vulnerable secondary and/or tertiary amine functional groups [26, 27]. It is discovered that nitrosamine impurities were found in sartan‐class drugs containing a tetrazole ring, as well as in metformin and ranitidine [23]. For sartan‐class drugs, such as valsartan, losartan, and irbesartan, the formation of tetrazole ring during the drug synthesis process presents a risk of nitrosamine contamination. The use of sodium nitrite (NaNO_2_) as quenching agent and dimethylformamide (DMF) as a solvent would cause degradation, resulting in the formation of dimethylamine (DMA) [28]. In an acidic environment, a secondary amine molecule in DMA will interact with nitrite to generate a nitrosamine compound like NDMA [29, 30].

Excipients have also been found to contribute to the formation of nitrosamines in drugs. For example, in solid dosage forms, nitrite is primarily associated with excipients that present at the highest formulation percentages (e.g., diluents), resulting in an average nitrite content of approximately 1 μg/g in a typical formulation [25]. Prior research reveals that excipients with low nitrite levels (LNLs) are desirable, as they can significantly reduce the risk of nitrosamine production and help maintain impurity levels within acceptable limits, even when changes in excipient composition occur [31]. Additionally, inappropriate monitoring of residual solvents would also influence the presence of nitrosamine substances in drugs [32].

The lidding foil used for blister packaging is usually coated with nitrocellulose. This substance has been found to decompose into nitrosating agent (NO_x_) at ambient temperature, while printing inks may also contain low‐molecular‐weight secondary amines, particularly dimethylamine and diethylamine [33]. Nitrosamine substance in the lidding foil can volatilize during the heat‐sealing process, thus the dispersed vapor settles onto unsealed drug products in blister cavities prior to sealing [34]. Furthermore, storage temperature should be monitored wisely because it may impact the nitrosamine formation [35]. A previous study has reported that ranitidine, which contains both nitrite and dimethylamine moieties, may show increased NDMA accumulation when stored at elevated temperatures [36].

The variety of nitrosamine formation pathways gives rise to structurally distinct classes, which are commonly categorized as small‐molecule nitrosamines and nitrosamine drug substance‐related impurities (NDSRIs). Small‐molecule nitrosamine impurities could be present in a wide range of pharmaceutical products. For instance, these include NDMA, *N*‐nitrosodiethylamine (NDEA), *N*‐nitrosomethylphenylamine (NMPA), *N*‐nitrosodiisopropylamine (NDIPA), *N*‐nitrosoisophenylethylamine (NIPEA), *N*‐nitrosodibutylamine (NDBA), and *N*‐nitroso‐N‐methyl‐4‐aminobutyric acid (NMBA) [15]. On the other hand, NDSRIs are typically specific to each API and share structural similarities [15]. The primary mechanism of NDSRI formation is based on the nitrosation of amine groups (secondary, tertiary, or quaternary) that are inherently attached to the active substance or its fragments when exposed to nitrosating agents, such as nitrite impurities in excipients [24]. Due to the unique characteristics of NDSRIs, product‐specific analytical method development is required for running analysis.

### 2.2. Analytical Challenges in Pharmaceutical Matrices

A key challenge in nitrosamine analysis is achieving trace‐level detection. Analytical methods must reliably detect and quantify nitrosamines at ng/g or ng/mL concentrations. This is necessary because very low permitted intake limits require adequate precision and accuracy. The complexity of the sample matrix differs significantly between APIs and finished drug products. APIs typically present simpler matrices. Finished drug products often include excipients, coatings, colorants, sweeteners, and degradation products. These additional components may interfere with analyte extraction, chromatographic separation, or detection. The existence of components other than nitrosamines may cause matrix effects. Such matrix effects can suppress or enhance detector signals, thereby compromising analytical accuracy [37].

Furthermore, another challenge comes from the structural diversity of nitrosamine impurities. Small molecular nitrosamines have low molecular weights and are relatively volatile [15]. Short nitrosamine compounds, such as NDMA and NDEA, have high solubility in water, while the solubility of NDPA and NDBA is lower [38]. In contrast, larger molecules, such as NDSRIs, tend to exhibit solubility limitations due to their higher molecular weights and more complex structural features, resulting in distinct physicochemical properties [39]. These variations affect extraction behavior, chromatographic retention, and detector compatibility.

Appropriate sample preparation is therefore crucial to improve analyte recovery and prevent matrix interference. Collectively, these challenges demonstrate that no single analytical method is universally applicable to all nitrosamine impurities and pharmaceutical matrices. Method selection should therefore be based on analyte characteristics, sample composition, required sensitivity, and laboratory capabilities. These considerations underscore the necessity for analytical methods specifically tailored to the properties of nitrosamine impurities and pharmaceutical matrices.

## 3. Analytical Methods for Nitrosamine Determination

### 3.1. Regulatory Official Methods

The presence of nitrosamine impurities in long‐term medications, such as antihypertensives and antidiabetic medications, poses a significant concern for patients consuming these drug products daily [35, 40]. Therefore, routine quantification of nitrosamines in APIs and drug products should be implemented by pharmaceutical manufacturers to ensure patient safety. In this context, regulatory agencies and pharmacopeial bodies have established official methods for nitrosamine determination, as shown in Table 2.

**TABLE 2:** Regulatory approaches for nitrosamine analysis in pharmaceuticals.

Regulatory body	Nitrosamines analyzed	Method	Reference
US Pharmacopeia	NDMA, NDEA, NDIPA, NEIPA, NMBA, NDBA	HPLC–HRMS, GC–MS, HPLC–MS/MS, GC–MS/MS	[14]
The European Pharmacopoeia	NDMA, NDEA, NDBA, NMBA, NDiPA, NEiPA, NDPA	GC–MS, LC–MS/MS, GC–MS/MS	[41]
SWISS MEDIC	NDMA, NDEA, EIPNA (ethylisopropylnitrosamine), NDIPA (*N*‐nitroso‐di‐iso‐propylamine), DPNA (*N*‐nitrosodi‐n‐propylamine), DBNA (*N*‐nitrosodi‐n‐butylamine)	GC–MS/MS	[42]
Government of Canada	NDMA and NDEA in ARBs, NDMA in ranitidine, NDMA in metformin	GC–MS/MS (direct injection), UPLC–MS/MS	[43]
Taiwan Food and Drug Administration	NDMA, NDEA, NMBA, NDBA, NMEA, NDiPA, NDPA, NDiBA, NPIP, NMOR, NDiNA, NDCHA, NDPhA, *N-*nitroso in salbutamol, *N-*nitroso in fluoxetine, *N-*nitroso in paroxetine, *N-*nitroso in duloxetine, *N-*nitroso in metformin	LC–MS/MS, GC–MS/MS	[44]
CVUA Germany	NDMA in valsartan and ranitidine	LC–MS/MS	[45, 46]
Health Sciences Authority (HSA) Singapore	NDMA, NDEA	LC–MS/MS	[47]

As illustrated in Table 2, GC–MS/MS and LC–MS/MS are predominantly employed by regulatory authorities. This trend suggests that tandem MS currently represents the most practical balance between sensitivity, selectivity, and regulatory applicability. However, the analytical scope differs among agencies, and official methods are continually updated as new nitrosamines, especially NDSRIs, are discovered. Therefore, although these methods provide essential regulatory benchmarks, practical method selection must also consider analyte properties, matrix complexity, and laboratory capabilities.

### 3.2. Gas Chromatography (GC)–Based Method

The principle of gas chromatography includes the separation of compounds through a column by a carrier gas (e.g., helium, hydrogen, or nitrogen) [48, 49]. In this method, a solid or liquid sample is vaporized into a gas phase, followed by separation based on volatility and interaction with the stationary phase [50]. GC is suitable for compounds with low molecular weight, high volatility, and thermal stability, such as NDMA and NDEA [51–53]. The comparison between the studies of nitrosamine impurity analysis is shown in Table 3.

**TABLE 3:** The comparison between studies using gas chromatography.

Analyte	Sample matrix	Analytical method	Sample introduction/preparation	Estimated chromatographic run time	LOD	LOQ	Recovery (%)	RSD (%)	Ref.
NDMA NDEA	Sartans and metformin API and finished product	GC–MS/MS (ESI)	HS	25.8 min	NDMA: 0.2 μg/kg NDEA: 0.08 μg/kg	NDMA: 1.0 μg/kg NDEA: 0.2 μg/kg	NDMA: 95–105 NDEA: 93.6–105	NDMA: 0.4–3.5 NDEA: 0.4–3.3	[54]

NDMA	Ranitidine	HS–SPME–GC–MS (ionization using APCI)	HS–SPME	16 min	1 μg/L	5 μg/L	NA	NA	[55]

NDMA NDEA EIPNA DIPNA	Losartan potassium API	HS–GC–MS	Headspace	15.5 min (+1.7 min cool down period)	5 ppb (NDMA and NDEA) 25 ppb (DIPNA and EIPNA)	25 PPB (NDMA and NDEA) 50 ppb (DIPNA and EIPNA)	Intraday: −4.19–4.89 Interday: −2.57–5.57	Intraday: 1.3–4.8 Interday: 1.3–5.9	[56]

NDMA NMEA NDEA NPYR NMOR NDPA NPIP NDBA NDPhA	Ranitidine tablet (150 and 300 mg per tablet)	GC–MS	Dispersive liquid–liquid microextraction	20 min	0.07–6.6 ng/g	0.21–21 ng/g	Lowest level: 80.2–102 (*n* = 36) Highest level: 94.7–105 (*n* = 36)	Repeatability (intraday, *n* = 9): 3.7–10 Reproducibility (interday, *n* = 27): 4.3–12	[57]

NDMA NMEA NDEA NDBA NMOR NPYR NPIP NDPA *N-*methyl‐npz	Cilostazol, sunitinib malate, and olmesartan medoxomil.	GC–MS (single quadrupole, electron ionization)	Centrifugation	24 min	0.15–1.00 ng/mL	0.45–3.00 ng/mL	Sunitinib malate: 94.09–111.22 Cilostazol: 95.91–104.87 Olmesartan medoxomil: 92.20–108.72	Repeatability: 0.76–5.75 (all nitrosamines) Intermediate precision: 1.13–7.65 System precision: 1.03–3.65	[58]

NDMA NDEA NMEA NEIPA NDiPA NDPA NDiBA NDBA NPIP NPYR NMOR NDiNA NDCHA, NDPhA	44 API (tetrazole‐ring compounds, antibiotics, antihistamines, antipsychotics, others)	GC–MS/MS	Direct injection and HS‐SPME	Direct injection: 14.5 min HS‐SPME: 12.3 min	NA	NA	Direct injection: 81.0–109.1 HS‐SPME: 95.7–102.2 (NDiNA, NDCHA, and NDPhA were not detected owing to the inadequate repeatability)	Direct injection: 0.2–15.1 HS‐SPME: 2.9–9.1	[59]

NDMA NDEA NEIA NDIPA	Valsartan API	GC–MS/MS	HS	15.5 min	NDMA: 0.03 ppm NDEA: 0.02 ppm NEIA: 0.03 ppm NDIPA: 0.03 ppm	NDMA: 0.09 ppm NDEA: 0.06 ppm NEIA: 0.09 ppm NDIPA: 0.09 ppm	Mean % recovery at 50% level: 91.93 ± 1.75–114.42 ± 0.31 Mean % recovery at 100% level: 87.68 ± 0.76–113.62 ± 0.99 Mean % recovery at 150% level: 101.64 ± 0.49–122.75 ± 0.26	Interday: 1.45–6.38 Intraday: 2.88–9.15 Intermediate precision: 2.8–3.7	[60]

NDMA	Metformin HCl drug product	FE‐SH–GC–NPD	Full evaporation static headspace (FE‐SH)	11.25 min	S/N ratios: 16–24 for 2.4 ppb NDMA	S/N ratios: 20–83 for 4.8 ppb NDMA	Mean % recovery at each concentration level: 85–108	Repeatability: 4–5 Intermediate precision: %RSD for %recovery from 18 pooled results from both analysts: 7–8	[61]

NDMA NMEA NDEA NDPA NPYR NPIP NDBA	Metformin HCl tablet	HS–GC–IMS	HS	11.84 min	0.05–0.51 ng/mL	0.16–1.70 ng/mL	84.31–106.6	Intraday: 2.10–4.76 Interday: 6.50–12.93	[62]

NDEA NDMA NDIPA NMPA NEIPA NDBA	Drug product A, B, and C (not specified)	HS–GC–MS/MS	HS	NA	Product A: 0.10–0.75 ppm Product B: 3.9–29.9 Product C: 0.002–0.02	Product A: 0.32–2.49 ppm Product B: 13.0–99.7 Product C: 0.01–0.06	79.5–122.4	Repeatability: 0.49–3.29 Intermediate precision (*n* = 6): 0.12–2.26	[63]

Sample preparation plays a pivotal role in GC‐based nitrosamine analysis, particularly in GC–MS, where selecting an appropriate sample introduction method with minimal sample preparation is crucial to ensure method sensitivity and analyte recovery. Among the reported approaches, headspace (HS) sampling is one of the most frequently employed methods because it selectively transfers volatile analytes into the gas phase while also reducing nonvolatile compounds [64]. This minimizes solvent usage, lowers inlet maintenance, and improves system robustness when mass spectrometry (MS) is used as a detector [56, 60, 65–67]. In addition, HS‐based methods also give lower analytical costs [68]. HS can be performed in either static or dynamic mode, depending on whether analytes are equilibrated in the vial or continuously purged and trapped prior to injection [69].

HS technique can be combined with solid phase microextraction (SPME) to provide enrichment in analytes with weaker partitioning. When analyzing heat‐labile samples, such as ranitidine, the SPME technique can effectively eliminate high temperature usage, which is a hurdle in the analysis of ranitidine by GC [55]. Another comparison study also reported that the usage of HS‐SPME methods shows lower LOQ when analyzing 14 nitrosamines in pharmaceutical matrices compared to direct injection [59]. Another approach for sample introduction, such as dispersive liquid‐liquid microextraction (DLLME), can concentrate trace‐level nitrosamines in a pharmaceutical product [57].

GC can be coupled with several detection systems, including flame ionization detector (FID), nitrogen phosphorus detector (NPD), electron capture detector (ECD), and MS [70]. In pharmaceutical nitrosamine analysis, choosing a detector mainly depends on the need for very sensitive detection and strong selectivity, especially when working with complex samples. Concerning trace impurities like nitrosamines, coupling GC with MS is advantageous due to the selectivity and efficiency for simultaneously detecting multiple components [71]. This trend is reflected in recent studies, where tandem MS is more frequently employed than standard GC detectors due to its lower detection limits and less matrix interference. If the instrumental analysis run time is a key consideration in selecting an analytical method, a detector such as NPD or ion mobility spectrometry (IMS) may be a suitable option, as both offer chromatographic run times of less than 12 min [61, 62].

GC can give advantages, including shorter run time, straightforward operation, and lower solvent consumption and disposal compared to LC [72, 73]. However, its application may be less suitable for analytes with low volatility, high molecular weight, or a lack of thermal stability.

### 3.3. Liquid Chromatography (LC)–Mass Spectrometry

Among the available methods, LC coupled with MS (LC–MS) has become one of the most widely adopted methods for determining nitrosamine impurities, particularly by regulatory agencies. This is largely because of its ability to analyze compounds that are nonvolatile, thermally unstable, or structurally complex, which are generally not suitable for GC‐based methods. In LC–MS, after the chromatographic separations run, MS will ionize analytes in the ion source, generating charged fragments that are separated by the mass analyzer based on the mass‐to‐charge ratio (m/z). A combination of LC and MS provides high‐resolution separation with sensitive and selective detection capabilities [74]. The comparison of each studies using LC‐MS method is presented in Table 4.

Akin to GC‐based approaches, nitrosamine impurities require extraction from the API and drug products to improve analyte recovery, minimize matrix effects, and enhance method sensitivity and accuracy during analysis. The complexity of the pharmaceutical matrix significantly influences the selection of an appropriate extraction method. In oral solid dosage forms, excipients such as high‐molecular‐weight polymers may complicate extraction by promoting the adsorption or co‐extraction of interfering substances, which can compromise method robustness [75]. Therefore, optimized extraction and cleanup procedures are essential for reliable quantification of nitrosamines in these complex matrices [76]. In contrast, parenteral formulations, which are typically aqueous or buffered systems, present less complex matrices. Nevertheless, inorganic salts can interfere with ionization during MS, making preliminary separation with appropriate organic solvents necessary [77].

Various extraction methods have been applied depending on the sample type and its complexity. Simple approaches, such as centrifugation, are commonly used for sartan‐class drugs due to their simplicity and rapid separation efficiency [78–80]. For more complex matrices, solid phase extraction (SPE) is chosen because it can effectively clean complex samples and reduce matrix interference [13, 81]. Recent advancements in microextraction techniques, such as in‐tube solid‐phase microextraction (IT‐SPME) and dispersive inclusion complex microextraction (DICM), have improved extraction efficiency and minimized solvent consumption [82, 83]. Notably, application of IT‐SPME is considered an environmentally friendly option due to the ability to minimize the labor‐saving and usage of harmful organic solvents [84], whereas DICM improves the efficiency and extraction yield for less hydrophobic compounds through inclusion complex formation [82]. At last, liquid–liquid extraction (LLE) is a flexible method because it is simple and can be used in many situations [85]. However, this latter approach usually has lower selectivity and needs more solvent than other techniques.

Despite the application of appropriate sample preparation strategies, residual matrix components may still co‐elute with target analytes, thereby affecting ionization efficiency. On account of that, ionization is essential for using a mass spectrometer as a detector. Among the available ionization methods, electrospray ionization (ESI) and atmospheric pressure chemical ionization (APCI) are the most widely used for nitrosamine determination. Therefore, the selection between APCI and ESI majorly depends on analyte polarity and the existence of suitable moieties for ionization [86]. Particularly, APCI is more suitable for low to medium polarity analyte while ESI is more suitable if the nitrosamine analyte is more polar [81, 85, 87–89].

In addition to the ionization procedure, the selection of mass spectrometric acquisition mode plays a crucial role in achieving the required selectivity and sensitivity. Most LC–MS methods employ multiple reaction monitoring (MRM) mode using a triple quadrupole MS/MS [90]. In this approach, fragmenting a selected precursor ion into characteristic fragment ions [91] enables highly selective detection of a specific analyte. Particularly, for routine analysis application, MRM mode showed excellent sensitivity, specificity, and fast scanning rate [92]. Compared to the selected ion monitoring (SIM), which utilized only a single quadrupole [90], MRM offers strong selectivity by filtering both precursor and product ions, which greatly lowers background interference. Because of this, MRM works well for measuring trace amounts of nitrosamines in complex pharmaceutical samples. Furthermore, SIM is usually employed when it needs to enhance the detection of low‐intensity ions, as shown by high signal‐to‐noise ratios (S/N) and measurement precision [93].

This shows that LC–MS/MS is a reliable method for analyzing traces of nitrosamine impurities in API and drug products, including when the analyte is measured simultaneously. However, matrix effects during ionization can contaminate the equipment, which influences MS detection by reducing the sensitivity and quantification accuracy of the target analytes [94]. MS is also expensive compared to other detectors, such as UV‐Vis [95], and requires an isotopically labeled internal standard that costs more [96], thereby impacting the cross‐signaling [97].

**TABLE 4:** The comparison between studies using liquid chromatography coupled with mass spectrometer detector.

Analyte	Sample matrix	Analytical method	Sample introduction/preparation	Estimated chromatographic run time	LOD	LOQ	Recovery (%)	RSD (%)	Quantitation ion (m/z)	Ref.
NDMA NMEA NPYR NMBA NDEA NEIPA NDIPA NDPA NMPA NDBA	Metformin drug product	LC–MS/MS (HESI and APCI)	Mechanical shaking followed by cold centrifugation	13.5 min	NDMA: 0.1 ng/mL NMEA: 0.02 ng/mL NPYR: 0.04 ng/mL NMBA: 0.02 ng/mL NDEA: 0.04 ng/mL NEIPA: 0.02 ng/mL NDIPA: 0.1 ng/mL NDPA: 0.1 ng/mL NMPA: 0.04 ng/mL NDBA: 0.02 ng/mL	NDMA: 0.1 ng/mL NMEA: 0.04 ng/mL NPYR: 0.04 ng/mL NMBA: 0.04 ng/mL NDEA: 0.1 ng/mL NEIPA: 0.04 ng/mL NDIPA: 0.2 ng/mL NDPA: 0.2 ng/mL NMPA: 0.1 ng/mL NDBA: 0.1 ng/mL	86–112	3.2–13.4	NDMA: 75.055 ⟶ 58.054 NMEA: 89.071 ⟶ 61.071 NPYR: 101.071 ⟶ 55.071 NMBA: 147.076 ⟶ 117.125 NDEA: 103.087 ⟶ 75.113 NDIPA: 131.118 ⟶ 89.125 NDPA: 131.118 ⟶ 89.125 NMPA: 137.1 ⟶ 66.1 NDBA: 159.149 ⟶ 103.054	[98]

NDMA NDEA NMBA NEIPA	Valsartan, losartan potassium, irbesartan	HPLC–APCI–MS/MS	Centrifugation	Stop time: 5 min Post time: 3.5 min	0.2 ng/mL	0.4 ng/mL	NDMA: 96.6–98.3 NDEA: 90.3–101.1 NMBA: 98.3–100.7 NEIPA: 90.7–97.2	NDMA: 4.06 NDEA: 6.87 NMBA: 1.86 NEIPA: 6.57	NDMA: 75.1 ⟶ 58.1 NDEA: 147.1 ⟶ 117.1 NMBA: 103.1 ⟶ 29.1 NEIPA: 117.1 ⟶ 75.1	[79]

NDMA NMEA NDEA NPYR NMOR NDPA NPIP NDBA	Sartans	UPLC–APCI–MS/MS	Centrifugation	8 min	0.32–1.58 ng/mL	1.09–4.74 ng/mL	Intraday API: 86.4–107.8 DP: 82.1–114.1 Intraday API: 95.6–109.6 DP: 92.5–108.2	Intraday API:7.6–9.1 DP: 6.0–4.95 Interday API: 7.45–9.0 DP: 7.8–10.5	NDMA: 75.10 ⟶ 58 NMEA: 89.1 ⟶ 61 NDEA: 103.1 ⟶ 74 NPYR: 101.1 ⟶ 55 NMOR: 117 ⟶ 87.2 NDPA: 131 ⟶ 43.1 NPIP: 115.1 ⟶ 41.2 NDBA 159.2 ⟶ 57.1	[99]

NDMA NDEA NDIPA NEIPA NDBA	Valsartan tablet (NDMA, NDEA, NDIPA, NEIPA, NDBA) Losartan tablet (NDMA, NDEA, NDIPA, NEIPA, NDBA) Ranitidine injection (NDMA, NDBA)	UPLC–APCI–MS/MS	Dispersive inclusion complex microextraction (DICM)	11 min	0.011–0.018 ng/mL	0.0037–0.5 ng/mL	NDMA: 96.2–104.4 (valsartan), 101.1–104.8 (losartan), 96.5–103.0 (ranitidine), 99.9–100.3 (standard) NDEA: 97.7–104.1 (valsartan), 96.4–99.5 (losartan), 97.7–104.5 (ranitidine), 99.8–102.0 (standard)	NDMA: 4.7–5.2 (valsartan), 3.8–4.2 (losartan), 3.9–5.4 (ranitidine), 2.7–3.2 (standard) NDEA: 4.7–5.8 (valsartan), 4.6–5.1 (losartan), 5.0–5.3 (ranitidine), 2.9–3.5 (standard)	NDMA: 75 ⟶ 43 NDEA: 102.9 ⟶ 75.0 NDIPA: 131.015 ⟶ 42.91 NEIPA: 116.935 ⟶ 74.98 NDBA: 159.046 ⟶ 56.99	[82]

NMBA	Candesartan cilexetil, olmesartan medoxomil, irbesartan, valsartan	LC–APCI–MS/MS	Vortex and Centrifugation	8 min	3.0 ng/mL	0.9 ng/mL	89.9–115.7	%RSD intraday: 2.2–4.5 %RSD interday: 3.6–5.0	147.1 ⟶ 117.0	[80]

NDMA NDEA NDPA NDBA NPYR NPIP NMOR	Metformin	LC–ESI–MS/MS	IT‐SPME	16 min	NDMA, NDEA: 2.5 ng/mL NDPA, NDBA, NPYR, NPIP, NMOR: 0.5 ng/mL	NA	Intraday: 95.8–117.0 Interday: 93.3–114.9	Intraday: 2.5–5.5 Interday: 3.0–7.0	NDMA: 74.9 ⟶ 58.2 NDEA: 103.0 ⟶ 75.0 NDPA: 131.1 ⟶ 89.1 NDBA: 159.3 ⟶ 103.1 NPYR: 101.1 ⟶ 55.2 NPIP: 115.1 ⟶ 68.9 NMOR: 117.0 ⟶ 87.1	[83]

NTTP	Sitagliptin tablet	LC–MS (QDa)	Centrifugation	25 min	0.15 ng/mL	0.5 ng/mL	87.78–99.96	Precision: 0.5 Repeatability: 4.6	222	[100]

NDMA NMBA NDEA NEIPA NDIPA NDPA NMPA NMBA	Valsartan API	UFLC–APCI–MS/MS	NA	20 min	0.06 ppb	NDMA: 0.25 ppb NDEA, NDP, NEIPA, NDIPA, NMBA, NMPA: 0.15 ppb	90.71 ± 0.57–121.05 ± 0.65	1.66–7.63	NDMA: 75.05 ⟶ 43.03 NMBA: 147.08 ⟶ 44.05 NDEA: 103.09 ⟶ 47.02 NEIPA: 117.08 ⟶ 75.05 NDIPA: 131.12 ⟶ 43.05 NDPA: 131.12 ⟶ 43.05 NMPA: 137.1 ⟶ 66 NDBA: 159.15 ⟶ 57.07	[101]

NDMA NMBA NDEA NEIPA NDIPA NDBA	Azilsartan, valsartan, telmisartan, olmesartan, losartan, irbesartan	LC–APCI–MS/MS	Centrifugation	20 min	S/N: 3.9–7.7	S/N: 15.7–37.2	Within 10%	< 6 for all six impurities	NDMA: 75.0 ⟶ 43.1 NMBA: 147.0 ⟶ 117.0 NDEA: 103.0 ⟶ 75.0 NEIPA: 117.0 ⟶ 47.1 NDIPA: 131.0 ⟶ 43.0 NDBA: 159.1 ⟶ 57.0	[102]

NDMA NDEA	Irbesartan, olmesartan, metformin (all in tablet dosage form)	UPLC–APCI–MS/MS	Centrifugation	10 min	NDMA: 0.35 ng/mL NDEA: 0.29 ng/mL	NDMA: 0.55 ng/mL NDEA: 0.37 ng/mL	NDMA: 96.0–98.4 NDEA: 96.0–98.0	NDMA: 3.46 NDEA: 2.69	NDMA: 75 ⟶ 58.1 NDEA: 103 ⟶ 75	[76]

NTTP	Sitagliptin phosphate (tablet) and sitagliptin phosphate monohydrate (API)	LC–MS/MS (ESI)	NA	10 min	NA	DP: 0.056 ng/mL Nitrite salt: 0.21 ng/mL	DP: 95.5–105.2 API: 96.2–104.4 Nitrite salt: 87.6–107.8	DP: 3.4 API: 2.6	222.05 ⟶ 192.13	[87]

NDMA NMBA NDEA NEIPA NDIPA NDPA NMPA NDBA NNP NNPL 1M4NP 2,4NPE 2,4NP1P 4MN1,3PB NMP	Valsartan, losartan potassium, candesartan cilexetil, olmesartan medoxomil, telmisartan, irbesartan	LC–MS/MS	Simple dissolution in a solvent and SPE (comparison)	32 min	NA	0.01 ppm	At 0.01 ppm: 94.4–119.5 At 4.5 ppm: 78.5–104.2	Repeatability: < 20% Reproducibility: 6 results ≤ 20.0% 12 results ≤ 25.0%	NDMA: 75 ⟶ 58 NMBA: 146.9 ⟶ 117 NDEA: 103 ⟶ 75 NEIPA: 117.1 ⟶ 75 NDIPA: 131.1 ⟶ 88.9 NDPA: 131.1 ⟶ 88.9 NMPA: 137 ⟶ 107 NDBA: 159 ⟶ 103 NNP: 114.98 ⟶ 68.94 NNPL: 100.97 ⟶ 55.07 1M4NP: 129.94 ⟶ 100.02 2,4 NPE: 160 ⟶ 99.02 2,4 NP1P: 194.1 ⟶ 95.97 4 MN 1,3PB: 208 ⟶ 121.8 NMP: 116.89 ⟶ 85.93	[13]

MeNP NDELA NDMA NMOR NMBA NPYR NMEA NDEA NPIP NEIPA NDIPA NMPA NDPA NDBA NDPhA	APIs [18] used: losartan valsartan irbesartan Olmesartan medoxomil Candesartan cilexetil Ranitidine Metformin Norfloxacin Tramadol Chloroquine Nifedipine Rivastigmine Timolol Raloxifene Oxytetracycline Ciprofloxacin Chlorpheniramine Rifampicin	LC–ESI–HRMS	SPE	12 min	NA	0.8–2.9 μg/L	85–115	< 10	MeNP: 130.0975 NDELA: 104.0710 NDMA: 75.0560 NMOR: 117.0660 NMBA: 145.0608 NPYR: 101.0713 NMEA: 89.0716 NkDEA: 103.0869 NPIP: 115.0867 NEIPA: 117.1024 NDIPA: 131.1180 NMPA: 137.0709 NDPA: 131.1180 NDBA: 159.1489 NDPhA: 199.0863	[81]

NDBA NMBA NMPA NDPA NEIPA NDIPA NDMA NDEA NNMP	Nebivolol tablet	Ultra‐sensitive LC– APCI–MS/MS	Centrifugation	70 min	3.25–6.48 ppb	9.85–19.62 ppb	At LOQ level: 85.43 ± 2.22–195.30 ± 1.91 At 50% level: 92.07 ± 1.83–119.23 ± 1.29 At 100% level: 100.80 ± 2.33–125.30 ± 1.13 At 150% level: 100.90 ± 1.61–126.73 ± 2.13	1,5‐3,4	NDBA: 159.1 ⟶ 41.1 NMBA: 147.1 ⟶ 44.2 NMPA: 137.1 ⟶ 66.1 NDPA: 131.1 ⟶ 43.1 NEIPA: 117.1 ⟶ 75.1 NDIPA: 131.1 ⟶ 89.1 NDMA: 75.1 ⟶ 43.1 NDEA: 103.1 ⟶ 75.1 NNMP: 130.1 ⟶ 58.2	[103]

MeNP NMBA NDMA NDEA NEIPA NMPA NDIPA NDBA	10 pharmaceutical products in various dosage forms	LC–ESI–MS/MS	LLE	12 min	0.05–0.8 ng/mL	0.1–2.0 ng/mL	Intraday: 91.5–108.7 Interday: 92.6–110.1	Intraday: 0.3–5.3 Interday: 1.3–9.0	NDMA: 75.1 ⟶ 43.1 NDEA: 103.1 ⟶ 75.2 NEIPA: 117.0 ⟶ 75.1 NDIPA: 131.1 ⟶ 43.2 NMBA: 147.0 ⟶ 117.0 MeNP: 130.1 ⟶ 58.2 NDBA: 159.2 ⟶ 57.2 NMPA: 137.0 ⟶ 66.1	[85]

NNor NHCT N Ena NSal, NFolAc NProp NMeto NMePh NBeta NBume NLor NFluo NVar	Nortriptyline HCl Hydrochlorthiazide Enalapril maleate Salbutamol hemisulfate Folic acid Propranolol hydrochloride Metoprolol tartrate Methylphenidate hydrochloride Betahistine hydrochloride Bumetanide Loratadine Fluoxetine Varenicline tartrate	LC–ESI–MS/MS	SPE	12 min	0.08–50 pmol/mL	0.16–250 pmol/mL	86.0–114.8	2.9–15.0	NNor: 293.1 ⟶ 233.1 NHCT: 324.9 ⟶ 281.1 N Ena: 406.1 ⟶ 116.1 NSal: 267.1 ⟶ 151.2 NFolAc: 469.1 ⟶ 439.1 NProp: 289.1 ⟶ 145.1 NMeto: 297.2 ⟶ 72.1 NMePh: 263.1 ⟶ 84.1 NBeta: 166.1 ⟶ 93.0 NBume: 392.1 ⟶ 318.1 NLor: ‐ NFluo: 339.1 ⟶ 117.1 NVar: 241.1 ⟶ 211.1	[88]

NDMA NDIPA NIPEA NMAP	Sitagliptin phosphate monohydrate API	LC–APCI–MS/MS	Sonication and dilution	18 min	NA	NDMA: 74.19 ng/g NIPEA: 196.62 ng/g NDIPA: 20.36 ng/g NMAP: 13.65 ng/g	90.23–103.36	NA	NDMA: 75.00 ⟶ 58.00 NDIPA: 131.16 ⟶ 42.98 NIPEA: 117.16 ⟶ 74.97 NMAP: 138.032 ⟶ 107.986	[104]

### 3.4. LC‐Based Method With UV or Fluorescence Detection

Despite the widespread use of LC–MS/MS for nitrosamine determination, the high cost of instrumentation, the use of isotopes as internal standards, and operational complexity may restrict its routine implementation in certain laboratories [105, 106]. Alternative LC‐based methods employing ultraviolet (UV) and fluorescence (FLD) detectors have been explored as a more practical and cost‐effective approach for routine analysis. UV‐based detection has promising applicability due to the fact that many nitrosamine compounds contain nitroso functional groups that act as chromophores. These functional groups can absorb radiation at UV wavelengths [107]. On the other hand, FLD offers enhanced sensitivity for selected derivatized nitrosamine compounds.

The primary obstacles associated with UV‐ and FLD‐based nitrosamine analysis include lower sensitivity for ultra‐trace quantification (ng/mL or lower), dependence on chromophoric or derivatizable moieties, and the lack of structural confirmation compared to MS‐based methods. Nevertheless, these detection techniques may still serve as potential alternatives for routine screening and quality control applications. This is because LC–MS/MS is not entirely free from analytical limitations. Technical concerns, such as matrix interference and ion suppression effects, may still occur, especially for compounds with m/z values less than 100 Da, such as NDMA with a molecular mass of 74 Da [108]. In addition, derivatization strategies have been reported to significantly improve detector response, thereby increasing the applicability of UV‐ and FLD‐based methods for trace analysis [109]. One of the major discoveries from Table 5 is that derivatization‐assisted fluorescence detection (FLD) methods often obtained lower detection limits than direct UV‐based approaches, highlighting the relevance of signal enhancement strategies for trace‐level nitrosamine research.

**TABLE 5:** The comparison between studies using liquid chromatography coupled with UV detector and FLD.

Analyte	Sample matrix	Analytical method	Sample introduction/preparation	Estimated chromatographic run time	LOD	LOQ	Recovery (%)	RSD (%)	Ref.
NDMA NDEA NDIPA	Losartan	LC‐UV (λ_abs_ = 230 nm)	Centrifugation and filtration	30 min	NDMA: 0.011 μg/mL NDEA: 0.017 μg/mL NDIPA: 0.018 μg/mL	NDMA: 0.021 μg/mL NDEA: 0.025 μg/mL NDIPA: 0.028 μg/mL	NDMA: 93.2–100.1 NDEA: 85.8–96.4	NDMA: 2.18 NDEA: 4.29 NDIPA: 13.18	[110]

NDMA NDEA NEIPA NDIPA NDBA NMBA	Valsartan API and tablet	UFLC‐UV (λ_abs_ = 228 nm)	Dissolution and dilution	12 min	NDMA: 0.013 μg/mL NMBA: 0.011 μg/mL NDEA: 0.006 μg/mL NEIPA: 0.011 μg/mL NDIPA: 0.007 μg/mL NDBA: 0.011 μg/mL	NDMA: 0.041 μg/mL NMBA: 0.034 μg/mL NDEA: 0.020 μg/mL NEIPA: 0.035 μg/mL NDIPA: 0.022 μg/mL NDBA: 0.034 μg/mL	NDMA: 98.60–102.05 NMBA: 99.00–101.81 NDEA: 98.30–101.35 NEIPA: 98.60–102.84 NDIPA: 98.50–101.14 NDBA: 98.10–100.23	< 2 for all impurities in repeatability, intraday, and interday evaluation	[111]

NDEA	Losartan and valsartan, both are analytical standards and tablets	HPLC‐UV (PDA)	Dissolution and dilution	10 min	0.2 μg/mL	0.5 μg/mL	98.8 ± 0.4–102.2 ± 0.2	Intraday: 100.4 ± 0.3–101.9 ± 0.4 Interday: 100.7 ± 0.6–101.7 ± 1.0	[112]

NDMA NMEA NDEA NEIPA NDPA NDIPA NDBA NMBA	Atorvastatin calcium trihydrate and itraconazole	LC‐UV (λ_abs_ = 235 nm)	Combining drugs with formic acid, followed by API removal via crystallization	30 min	NA	NA	ATS: 45.2 ± 0.6–119.1 ± 8.5 ITC: 85.8 ± 2.3–109.6 ± 3.3	ATS: 0.78–25.09 ITC: 1.09–6.27	[94]

NDMA NDEA	Enalapril maleate	HPLC‐FLD (λ_ex_/λ_em_ dansyl chloride = 340/530 nm; λ_ex_/λ_em_ Fmoc‐Cl = 265/313 nm)	Denitrosation and pre‐column derivatisation using dansyl chloride and Fmoc‐Cl	25 min	NDMA: 0.013 μg/g NDEA: 0.017 μg/mL	NDMA: 0.038 μg/g NDEA: 0.050 μg/mL	NDMA: 74.2 ± 4.2–1011.6 ± 16.1 NDEA: 90.6 ± 2.9–125.4 ± 7.4	NDMA: 3.86–16.03 NDEA: 2.54–18.05	[108]

NDMA NDEA	Lisinopril API and lisinopril dihydrate tablets	HPLC‐FLD (λ_ex_/λ_em_ = 340/530 nm)	LLE, denitrosation, and precolumn derivatization using dansyl chloride	12.5 min	NDMA: 4.7 ng/mL NDEA:0.04 ng/mL	NDMA: 14.4 ng/mL NDEA: 0.13 ng/mL	NDMA: 91.9–108.4 NDEA: 90.3–108.4	NDMA: 1.4–9.2 NDEA: 4.5–13.4	[106]

NDMA NDEA	Reference standard, Uniphyllin^®,^ Xylozan^®^	HPLC‐FLD (λ_ex_/λ_em_ = 450/540 nm)	Extraction (comparison between LE and SPE) denitrosation and pre‐column derivatisation using NBD‐Cl	16 min	NDMA: 0.1 ng/mL (concentration range 0.5–10 ng/mL) and 0.3 ng/mL (concentration range 1.7–20 ng/mL) NDEA: 0.9 ng/mL (concentration range 5–100 ng/mL) and 3.0 (concentration range 16.7–200 ng/mL)	NDMA: 0.4 ng/mL (concentration range 0.5–10 ng/mL) and 1.3 ng/mL (concentration range 1.7–20 ng/mL) NDEA: 3 ng/mL (concentration range 5–100 ng/mL) and 10.0 (concentration range 16.7–200 ng/mL)	NDMA: 99–102 NDEA: 98.2–101.6	Repeatability: NDMA: 0.2–2.7 NDEA: 0.3–2.4 Intermediate precision: NDMA: 0.5–1.9 NDEA: 1.9–2.9	[113]

Several studies have examined LC‐based detectors for pharmaceutical substances and products, as summarized in Table 5. While all evaluated methods employed reversed‐phase LC, considerable differences were observed in the selection of the stationary phase, composition of the mobile phase, and elution procedures. Patil et al. and Bodiwala et al. used conventional C18 columns, whereas Yamamoto et al. applied a HyperCarb column (4.6 mm ID × 100 mm, 3 μm), characterized by chemical stability to withstand temperatures of approximately 200°C [114, 115]. HyperCarb is a porous graphitic carbon (PGC) column that gives complementary selectivity to conventional silica‐based (primarily C18) stationary phases, as used in a multidimensional separation method [116]. However, Yamamoto et al. found that the method needed 15 min to reach equilibrium before it could be reused. This timing may be important for future research. Furthermore, Patil et al. used isocratic elution, while the other two studies applied gradient elution with shorter overall performance time [117].

The wavelength in LC‐UV directly determines the sensitivity and selectivity of nitrosamine impurity detection. Based on the studies reviewed, all authors determined the analytical wavelength at 228–235 nm. Both the wavelength and intensity of the absorption were strongly dependent on the molecular structure of the analyte [118]. Patil et al. selected a detection wavelength of 230 nm using a diode array detector. Bodiwala et al. used 228 nm based on the presence of all impurities observed in the valsartan sample, alongside a photodiode array (PDA) detector, which enabled simultaneous detection at multiple wavelengths, compared to single‐element detectors [119]. Meanwhile, Yamamoto et al. used 235 nm as the detection wavelength for accurate UV measurement of nitrosamine and baseline stability in the chromatogram.

Like the discussion in the previous section, sample preparation is considered a substantial step before analysis starts. Yamamoto et al. used a distinctive method for sample preparation by combining drug products with formic acid to crystallize and remove API. This achieved over 99.96% removal with 0.5 mol/L ammonium acetate (AmAc) as a solvent, allowing for further analysis of the hydrophobic drug through LC‐UV. Meanwhile, Patil et al. found that dissolving losartan directly in methanol and diluting with water produced low response and broad peaks. To improve peak shape and response, losartan should be slurried in water, filtered, and diluted using methanol.

Compared to UV, the FLD‐based technique can provide a significant advantage, especially if the analysis is intended for more than just initial screening. Although it can be detected using UV spectra, the *N-*nitroso group exhibits low molar absorptivity, rendering direct HPLC‐UV analysis highly susceptible to poor detectability and inadequate sensitivity for trace‐level regulatory compliance [79]. Moreover, in complex drug product matrix, UV detection suffers from a critical lack of selectivity due to co‐elution and matrix interference [120]. For sample preparation, all studies use the LLE technique with dichloromethane (DCM) as a solvent. Nitrosamine is neutral and likely found in the DCM layer, while amine tends to be charged and remain in the aqueous layer during extraction [108].

For the detection of nitroso compounds using FLD, the nitroso group must be removed through denitrosation using a reagent such as HBr in acetic acid to produce a secondary amine, which latter reacts with a fluorescent reagent known as a derivatization reagent [106]. Alternatively, nitrosamine can be separated by HPLC, denitrosated with UV light, and derivatized for detection using a chemiluminescence detector [108]. After denitrosation, samples are incubated and heated to evaporate DCM and excess acids. After denitrosation, samples are incubated and heated to evaporate DCM and excess acids.

FLD analytical workflow typically incorporates a pre‐ or post‐column derivatization step. This step aims to improve the detection of analytes, alter the molecular structure or polarity of analytes, modify the matrix for better separation, and stabilize sensitive analytes [121]. Pre‐column derivatization involves adding the reagent before chromatographic separation, whereas post‐column derivatization is performed after separation but before detector analysis [121, 122]. From the studies abridged in Table 5, Tsanaktsidou et al. used Dansyl‐Cl as a derivatization reagent, Kamaris et al. used 4‐chloro‐7‐nitrobenzofurazane‐Cl (NBD‐Cl), while Boczar et al. compared between Dansyl‐Cl and Fmoc‐Cl. It is reported that the use of 9‐fluorenylmethyl chloroformate (Fmoc‐Cl) generated fewer interfering peaks compared to dansyl chloride, thereby improving analytical selectivity [108]. Fmoc‐Cl enables rapid derivatization of primary and secondary amines into stable derivatives, which are suitable for dual UV and FLD [123, 124]. Fmoc‐Cl derivatization may yield residual reagent‐ and byproduct‐related peaks, which might increase chromatographic background and complicate analysis, although no direct interference with nitrosamine analytes was observed [124]. In contrast, NBD‐Cl requires a longer reaction time due to its stability in aqueous solution [125]. Additionally, this reagent exhibits a unique “turn‐on” mechanism, remaining undetectable until it chemically binds to the denitrosated target. As a result, any excess reagent does not interfere with the analytical results [126]. Both Fmoc‐Cl and NBD‐Cl are sensitive derivatization reagents for detecting primary and secondary amines. The choice between them depends on the specific requirements of the analysis.

LC‐UV and LC‐FLD methods provide cost‐effective alternatives to LC–MS‐based techniques, particularly for laboratories with limited access to advanced instrumentation. Among these approaches, LC‐FLD methods offer better sensitivity for detecting low levels of nitrosamines. LC‐UV methods can still be used for routine screening and quality control when impurity levels are higher. However, limitations related to sensitivity, selectivity, and derivatization complexity remain significant considerations for their implementation in routine pharmaceutical analysis.

### 3.5. Other Methods

Beyond widely used chromatographic techniques such as LC and GC, additional analytical approaches have been explored to determine nitrosamine impurities in pharmaceutical products. These include total nitrosamine (TONO) assays, colorimetric, and chemiluminescence (CL), which provide valuable options for specific analytical challenges.

TONO is an approach used to quantify the total amount of nitrosamine impurities in a sample, rather than targeting individual compounds such as NDMA or NDEA. In this approach, the N‐NO bond in nitroso compounds is cleaved in the presence of denitrosating agents such as acidic triiodide (HI_3_) or UV irradiation, releasing nitric oxide (NO) or nitrite ions (${\text{NO}}_{2}^{-}$) [127–129]. Based on the molecules, nitrosamines can be quantified using colorimetric and chemiluminescence‐based detection methods [129]. Initially, TONO assays were more commonly applied in environmental and food analysis. Nonetheless, recent studies have further investigated their application as rapid pre‐screening tools for nitrosamine risk assessment in pharmaceutical substances and drug products. TONO assays are especially useful for detecting unknown or unexpected nitrosamine compounds in pharmaceutical samples. The quantification of apparent total nitrosamine content (ATNC) gives rapid and accurate results in a single readout using a thermal energy analyzer (TEA) as a detector [130]. Samples with ATNC values below the specified threshold are regarded as safe, whereas samples with positive ATNC results require further analysis using techniques such as GC–TEA to separate and quantify volatile nitrosamines [130].

Among TONO‐related detection systems, the chemiluminescence‐based detector is one of the most frequently used [131, 132]. Chemiluminescence (CL) is another analytical method, where chemical reactions generate electronically excited products that emit light [133, 134]. Pu et al. conducted a study using wastewater to compare the denitrosation mechanism between chemical treatment with HI_3_ and photolysis treatment at 254 nm wavelength [129]. The results showed that the HI_3_‐CL method was affected by matrix interferences, leading to higher TONO measurements in water than the UV‐CL methods [129]. In comparison, the UV‐CL method does not require chemicals, thereby reducing interferences from by‐products like NOI [135].

Moreover, the colorimetric method has shown promising potential for nitrosamine analysis by detecting the target analyte through a color change generated by chemical reaction, which can be used for qualitative or quantitative analysis [136]. This method relies on the Griess reaction between free nitrite (${\text{NO}}_{2}^{-}$) and azo compounds that produce colored pigments, whose absorbance is quantified [128, 137]. The analysis is carried out to analyze nitrosamine contaminants from food and beverages [138, 139] and water samples [128]. For example, Beard et al. developed a rapid and cost‐effective in‐field detection method for analyzing NDMA contamination in water using a colorimetric assay by photochemical nitrosation of a commercial naphtholsulfonate. The results showed that optimizing detection conditions, such as UV irradiation and response time, enhanced the colorimetric method, yielding an NDMA detection limit of 0.66 ppm.

Similar to TONO assay, since 1978, the WHO expert group has developed a nitrosation assay procedure (NAP) [140]. This test is designed to stimulate nitrosating conditions and evaluate the potential formation of NDSRIs as in rapid identification. In recent years, Sharma et al. have improved the standard NAP test protocol. In their method, different drugs are dissolved in an organic solvent and incubated at 37°C with tertiary butyl nitrite in a 1:10 M ratio, then analyzed by LC–MS. This approach yields higher nitrosation yields in organic solvents and reduces the required time, without creating any artifacts compared to the traditional NAP test [140]. Although NAP is a valuable tool, it serves as a predictive rather than a definitive analytical method. The simulated nitrosation conditions may not accurately reflect real‐world manufacturing, formulation, or storage environments, leading to an overestimation of nitrosation risk. Furthermore, NAP cannot substitute for confirmatory quantitative techniques such as LC–MS/MS or GC–MS/MS, which remain necessary for structural confirmation and precise quantification of nitrosamine levels in pharmaceutical products [58].

## 4. Comparison of Analytical Methods and Selection Considerations

Despite the methods presented above, no single analytical technique is universally suitable for all nitrosamine analysis. The selection of an analytical method should consider the physicochemical properties of the analyte, matrix complexity, required sensitivity, regulatory requirements, instrument availability, and operational cost. Consequently, understanding the comparative performance of currently available analytical methods is essential for selecting the most appropriate analytical strategy.

For routine quality control and early screening, simpler and less expensive analytical methods are often sufficient, especially if the target nitrosamines are present at higher levels [15]. However, confirmatory trace analysis and nitrosation risk assessment generally require more selective and sensitive instrumentation techniques that are proficient for distinguishing nitrosamine impurities from complex pharmaceutical matrices at trace levels. On the other hand, the nitrosation risk assessment has a wider objective, not only detecting the presence of nitrosamines but also evaluating the potential formation of NDSRIs under simulated nitrosating conditions [140].

Further consideration also depends on analyte volatility and thermal stability. The GC‐based method is more suitable for easily vaporized and thermally stable nitrosamines. The larger, less volatile, or thermally labile compounds are more compatible with an LC‐based system, which can avoid high temperatures and provide broader applicability for structurally complex analytes. Another factor in determining method selection is sensitivity requirements. Regulatory limits for nitrosamines are typically expressed as ng/g or ng/mL, making MS‐based techniques the preferred choice for confirmatory analysis. Nevertheless, for routine screening, LC‐UV and FLD methods remain valuable, especially when the laboratories have limited access to advanced MS instrumentation. The simpler operation and lower cost of these detectors promote their widespread adoption in routine pharmaceutical testing, although they exhibit reduced selectivity and limited ability to confirm molecular structures.

Moreover, the complexity of the matrix substantially affects method selection and the requirement of sample preparation. Pharmaceutical excipients such as salt, polymers, and also degradants from the products may contribute to matrix effects, ion suppression, or chromatographic interference [141]. For more complex samples, it often requires additional procedures of cleanup, such as SPE, SPME, or derivatization, to improve analyte recovery and analytical reliability [12, 37, 59]. Therefore, rather than directly favoring a single analytical platform, the choice of analytical method for nitrosamine determination should be viewed as a balance between analytical objective, analyte characteristics, sensitivity requirements, matrix complexity, and laboratory resources, as summarized in Figure 3.

**FIGURE 3:**
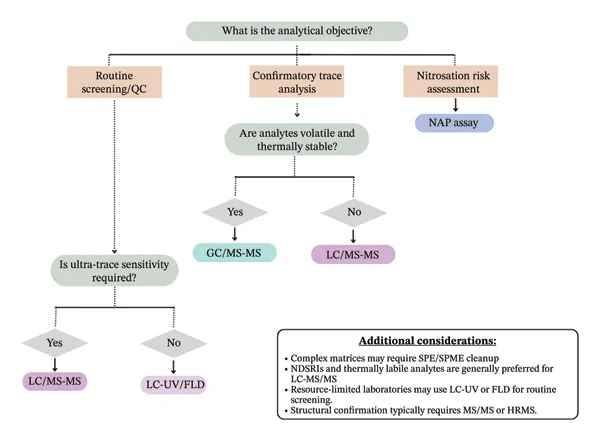
Decision tree for method selection.

## 5. Future Directions in Nitrosamine Analysis

Chromatography‐based methods are now the standard analytical approaches accepted by regulatory agencies around the world. However, some challenges remain, including matrix interference, complex sample preparation, high solvent use, and difficulties in identifying unknown nitrosamines. At the same time, growing global awareness of environmental sustainability has led to the adoption of green analytical chemistry (GAC) principles in pharmaceutical analysis [142, 143]. This concept is based on twelve principles of green analytical chemistry proposed by Galuszka et al. [144, 145] which intended to minimize waste formation, encourage energy efficiency, and reduce derivatization stages [146, 147].

One important application of GAC in nitrosamine analysis is the miniaturization of sample preparation workflows. These approaches offer minimal solvent utilization, minimal sample requirement, and lower energy consumption [143]. Junior et al. [143] have summarized several miniaturized sample preparation techniques in determining nitrosamines in pharmaceutical matrices, including solid‐phase microextraction (SPME), microextraction by packed sorbent (MEPS), disposable pipette extraction (DPX), and single‐drop microextraction (SDME). Although these extraction methods work in different ways, they all aim to use fewer hazardous solvents, save time, reduce sample handling, and make the analysis more sustainable. In the context of nitrosamine analysis, these approaches are particularly advantageous because regulatory monitoring often necessitates ultra‐trace detection across a wide range of pharmaceutical products [15]. Additionally, miniaturized extraction systems are increasingly compatible with automated and high‐throughput analytical workflows, which can enhance reproducibility and minimize analyst‐dependent variability [148]. However, further optimization is required to address challenges related to extraction robustness, matrix effects, and recovery consistency before these methods can be routinely implemented.

Besides improvements in analytical sustainability, future developments in nitrosamine analysis are also expanding analytical targets beyond conventional volatile nitrosamines [149]. Formerly, nitrosamine analysis primarily focused on small volatile nitrosamines. However, along with regulatory concerns have increasingly shifted toward NDSRIs [21], which possess greater structural diversity and more complex physicochemical properties. Recent studies have highlighted the importance of predictive nitrosation assessment to identify potential nitrosamine formation pathways before product commercialization [140, 150]. For instance, modified nitrosation assay procedures have been developed to simulate nitrosating conditions in pharmaceutical products, enabling rapid evaluation of the nitrosation susceptibility of APIs and related compounds [140].

In comparison with small nitrosamine molecules, NDSRIs may pose a greater analytical challenge due to their diverse chemical structure, broader polarity ranges, trace‐level concentrations, and limited availability of reference standards [151–153]. In particular, the scarcity of NDSRI analytical standards is a significant barrier to routine analytical implementation and method harmonization. Furthermore, unknown or newly formed nitrosamine structures may also exhibit unpredictable ionization behavior and fragmentation patterns during mass spectrometric analysis, complicating both structural elucidation and quantitative determination [154].

As a result, future analytical workflows may not rely only on conventional targeted quantification methods. Instead, nontargeted screening, suspect analysis, and predictive analytical strategies can facilitate the identification of previously unknown nitrosamine impurities and assess how they might form. These changes have sped up the use of advanced technologies, for instance, high‐resolution MS (HRMS), AI‐assisted screening tools, and automated high‐throughput systems for nitrosamine risk assessment. HRMS allows a full scan range for the detection of a broader range of nitrosamine impurities at trace levels [81], which enables nontarget identification of unknown nitrosamines. Concurrently, AI‐assisted screening and automated high‐throughput in silico systems can facilitate early nitrosamine risk assessment by detecting susceptible molecular structures and potential nitrosation pathways prior to impurity formation [155, 156].

## 6. Conclusion

In conclusion, several analytical methods are available for identifying nitrosamine impurities in drug substances and drug products. Among these, chromatographic‐based methods seem to be widely adopted in many situations and align with regulatory preferences. This is further supported by the variety of detectors that can be combined with chromatography systems, depending on the specific nitrosamine analyte to be identified. Notably, GC–MS and LC–MS remain dominant analytical platforms due to their high sensitivity, selectivity, and regulatory acceptance for trace‐level quantification. In addition to MS, various analytical techniques, such as LC‐UV, LC‐FLD, total nitrosamine assays, chemiluminescence, and colorimetric methods, also show potential for routine screening, especially when the laboratory is limited to MS facilities. Overall, the selection of analytical methods should be based on analyte characteristics, complexity of the matrix, required sensitivity, regulatory expectations, and the capability of laboratory. Therefore, analytical methods need to be continuously developed to support the dynamic analysis of nitrosamine impurities.

## Funding

This work was supported by Universitas Padjadjaran Research Grants through Review Article Grant 2025 No. 2078/UN6.O/TU.00/2025.

## Conflicts of Interest

The authors declare no conflicts of interest.

## Data Availability

Data sharing is not applicable to this article as no datasets were generated or analyzed during the current study.
